# Diagnoses of Mental Health Disorders Among Norwegian-Born Youth and Young Adults with Immigrant Parents—A Register-Based Study

**DOI:** 10.1007/s10903-025-01726-6

**Published:** 2025-07-09

**Authors:** Naima Said Sheikh, Melanie Lindsay Straiton, Guido Philipp Emmanuel Biele, Marte Karoline Råberg Kjøllesdal

**Affiliations:** 1 Norwegian University of Life Sciences, Ås, Norway; 2 Norwegian Institute of Public Health, Oslo, Norway

**Keywords:** Migration, Descendants, Mental health, Diagnosis, Youth and young adults

## Abstract

Adolescence and early adulthood are associated with an increase in mental health problems. Migration is also a risk factor for mental disorders. Yet, we know little about the risk of mental disorders among young descendants of immigrants. This study aims to investigate the risk of being diagnosed with a mental disorder between the ages of 16–30 years among those born in Norway with at least one immigrant parent compared to those with Norwegian-born parents. Data were sourced from the Medical Birth Registry of Norway, the Norwegian Patient Register, and Statistics Norway. Cox proportional hazard regressions were used to estimate hazard ratios (HR) of receiving diagnoses of depression, anxiety, bipolar affective disorders, eating disorders, and schizophrenia among those with one or two immigrant parents, in total and by parental region of origin. Individuals with Norwegian-born parents were the reference group. Analyses were adjusted for sex, year of birth, and parental education. Individuals with two immigrant parents had a lower hazard of most mental disorder diagnoses, including anxiety, depression, bipolar affective disorder, and eating disorders, compared to those with Norwegian-born parents, but a higher hazard of a schizophrenia diagnosis***.*** Individuals with one immigrant parent showed a higher hazard of mental disorder diagnoses compared to those with two Norwegian-born parents. These patterns were generally consistent across parental regions of origin. The findings show a lower risk of a diagnosis among those with two immigrant parents. It is unclear whether this is due to better health, different patterns of help-seeking or barriers to care. Higher risk of diagnoses among those with one immigrant parent points towards a need for targeted preventive measures.

**Supplementary Information:**

The online version contains supplementary material available at 10.1007/s10903-025-01726-6.

## Introduction

Mental health disorders pose substantial challenges to the overall well-being of youth and young adults worldwide. Globally, depression, anxiety, and behavioural disorders are among the leading causes of illness and disability among youth and young adults [1]. Suicide, which has a strong association with mental disorders, is globally the fourth, and in Europe, the second, leading cause of death among individuals aged 15–29 years old [1, 2]. In recent years, the relationship between migration and mental disorders, including among children of immigrants, has gained increasing attention [3–6]. The number of immigrants in Europe has increased substantially over the past few decades, with common reasons for immigration including labour migration, family reunification, and refugee resettlement. In Norway, immigrants make up 17.3% of the population, and 4.1% are their Norwegian-born offspring (often referred to as descendants) [7]. Many of these descendants are currently reaching youth and early adulthood, with parental origins most commonly from countries such as Poland, Lithuania, Pakistan, Iraq, Syria, Vietnam, Eritrea, and Somalia.

The literature lacks sufficient research on the mental health outcomes of descendants in their youth compared to peers with native-born parents. Some studies indicated that descendants of immigrants tend to experience higher levels of psychological distress and depression than native populations [8, 9]. In contrast, other studies have shown lower rates of psychiatric diagnoses given in healthcare services to descendants compared to native-born youth [10–13], while one study has found no differences between descendants and native background [14]. A recent review study among youth and young adults born in Nordic countries to immigrant parents showed that young people with immigrant parents were less likely to receive mental health diagnoses or to use health services for mental health challenges than others [15]. Given these mixed results, there is a need for more research that focuses on specific diagnoses, as several of the studies look at mental disorders as a whole [10–12] and from national studies that use register data over time.

There are many factors which could possibly contribute to differences in mental health between youth and young adults with immigrant parents and with native-born parents. These individual, social, and structural factors include emotional skills, relationships with friends and family, stressful life events, and socioeconomic position (SEP) [16, 17]. Previous studies have also highlighted stress related to acculturation, poor social support, and social exclusion and discrimination as significant determinants of mental health among immigrants [18–20]. Discrimination and experiences of not belonging are also commonly experienced by their descendants [21, 22], and may influence their mental health negatively. Furthermore, being raised by immigrant parents may also lead to tensions between the culture of the family and the majority society, which can increase the risk of poor mental health [11]. Individuals with one immigrant parent generally experience better socioeconomic conditions than those with two immigrant parents and benefit from the network and system knowledge of the native parent. However, potential diverging cultural expectations from family members may contribute to difficulties in defining one’s own identity and, in turn, mental health difficulties [11]. For young people in general, media influence, pressure to conform and exploration of identity represent challenges to mental health [1].

When experiencing mental health issues, youth and young adults with immigrant parents do not face the same structural barriers as their parents when seeking health care, such as language proficiency or lack of system knowledge. They may, however, still be influenced by different cultural perceptions of mental health, which can hinder both help-seeking and social support for help-seeking. The presence and magnitude of differences in risk of receiving mental disorder diagnoses in health care between different groups can indicate the extent to which such barriers exist. Documenting these differences may be an important step for planning measures to prevent and reduce social inequalities in mental health, as well as promoting good mental health in all groups. In Norway, a significant number of descendants have only recently reached young adulthood. Thus, the knowledge about the health of this group is limited. The current study aims to examine the risk of being diagnosed with different mental health disorders in specialist healthcare services between the ages of 16–30 years among those born in Norway with at least one immigrant parent compared to those born in Norway with two Norwegian-born parents, by parental region of origin and sex, adjusting for parental education as an indicator of SEP in childhood.

## Methods

### Data Sources and Variables

**Fig 1:**
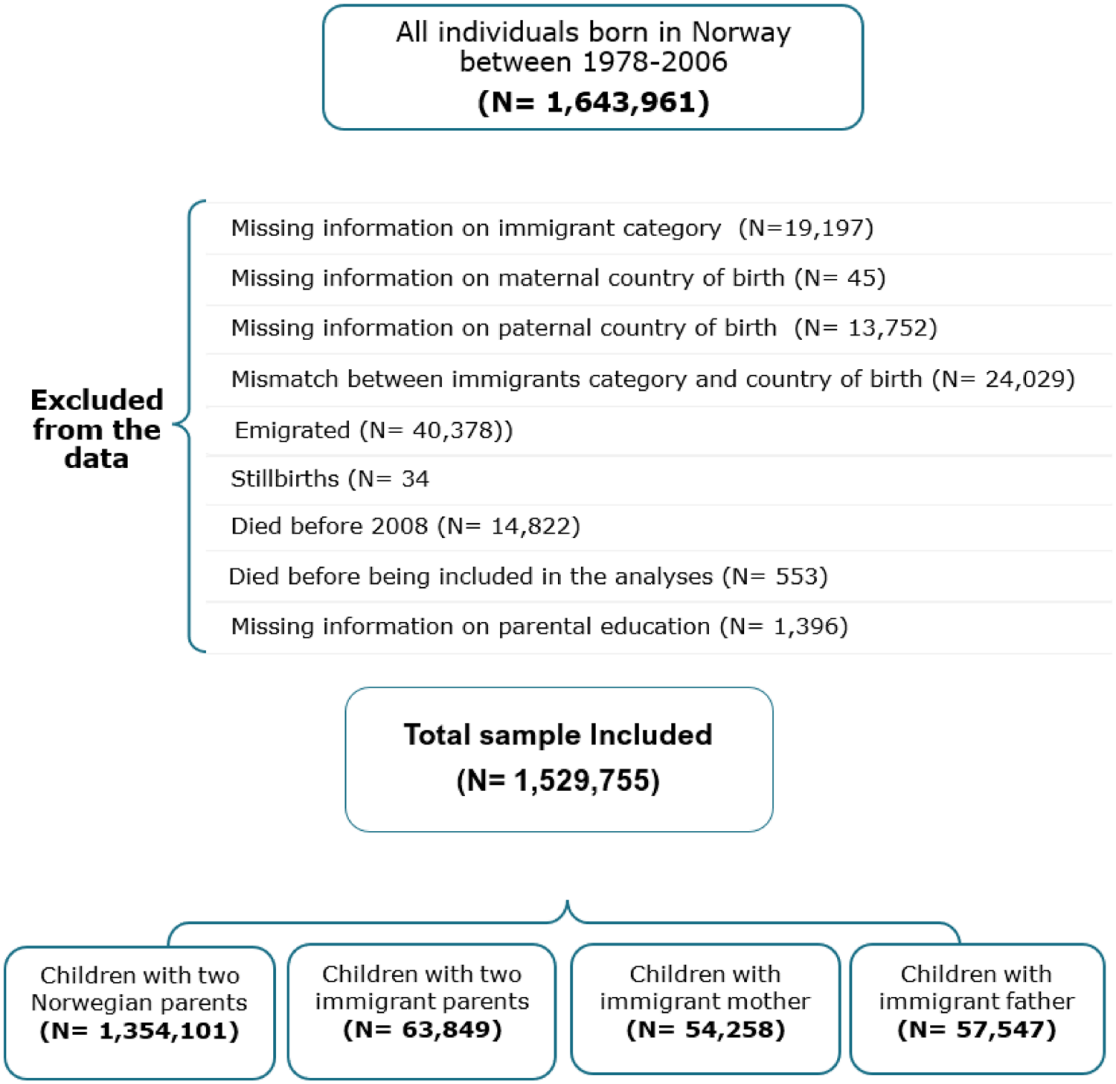
Flow chart of inclusion to analyses

Data from the Medical Birth Registry of Norway (MBRN) (which consists of data on the birth of everyone born in Norway since 1967), the Norwegian Patient Register (NPR) (includes diagnoses given in specialist health care from 2008 to 2022), Statistics Norway (information on migration and other sociodemographic data) and the Death Registry (date of death) were linked using a unique personal identification number. This study consisted of a population sample (N = 1,643,961), including individuals born in Norway between 1978 and 2006, to two Norwegian-born parents, to two foreign-born parents, or one Norwegian-born and one foreign-born parent. During the follow-up period, from 2008 to 2022, these individuals would have been turning 16–30. This age group was chosen because individuals aged 16 and above can visit healthcare services without needing to inform their parents. A low number of descendants in Norway are above the age of 30. We initially used the variable ‘immigration category’ from Statistics Norway, which indicates whether a person was born in Norway or not, and whether their parents (either one or both) were born in Norway or abroad, to identify our sample. Next, we used the variable parental country of birth to specify whether the mother or father was an immigrant among those with one immigrant parent, and to group into regions of origin Unfortunately, some individuals had missing data on mother or father’s country of birth in the register, which resulted in their exclusion from the study. As shown in Fig. 1, we excluded individuals with missing information on the immigration category (N = 19,197), individuals who had missing information on parental country of birth (N = 13,797) (missing mother: 45, missing father: 13,752), or where there was a mismatch between immigration category and parental country of origin (N = 24,029). We also excluded individuals who were registered as emigrated (N = 40,378), as the year of emigration was not available, children listed as stillborn (N = 34), those who passed away before 2008 (N = 14,822) or died before the inclusion in the analyses (N = 553), and those missing data on parental education (N = 1,396). This left us with a study sample of 1,529,755 individuals.

### Outcome Measure

The dependent variables were based on the following diagnoses (defined according to the International Classification of Disease, version 10 (ICD-10) and given in specialist healthcare (both inpatient and outpatient) from 2008 to 2022; (1) anxiety, obsessive–compulsive disorder, and adjustment disorder (F40-F43, coded as anxiety disorders), (2) depressive disorders (F32-F33), (3) bipolar affective disorder (F31), (4) schizophrenia and paranoid disorders (F20-F29), and (5) eating disorder (F50). Additionally, a dichotomous variable was created to indicate whether an individual had received any of these diagnoses.

### Explanatory Variables

*Year of birth* was coded into age groups: 1978–1982, 1983–1987, 1988–1992, 1993–1997, 1998–2002, and 2003–2006. *Sex:* Study participants were classified as male and female. *Parental education* was classified based on the highest educational level attained by the time the child was born by either parent and grouped into primary school (≤ 9 years), secondary school (10–12 years), low university level (13–16 years), and high university level (≥ 17 years).

*Parental region of origin:* Divided into five areas, according to national standards [7]: (1) EU, EEA, Oceania, USA, and Canada, (2) Europe (non-EU), (3) Asia, (4) Africa, and (5) Latin America. For those with two immigrant parents, their parental region of origin was based on the maternal country of birth, whereas for those with one immigrant parent, it was classified as the country where the respective immigrant parent was born. Immigrants are a highly heterogeneous group in terms of reasons for migration, culture, socioeconomic position, integration, and health. Although broad region groupings may mask many differences between groups in the region on all these dimensions, analysing the risk of receiving diagnoses by these groups may highlight some crucial differences between descendants with parents originating from different areas.

### Statistical Analyses

We calculated descriptive characteristics of the sample, including the number and percentage of individuals with each diagnosis, categorised by parental immigrant background. To assess differences in sociodemographic variables and diagnoses based on parental immigrant background, we performed Chi-square tests. We employed Cox proportional hazard regression models to calculate hazard ratios (HR) with 95% confidence intervals (CI) for each diagnosis, comparing individuals with one or two immigrant parents to those with a Norwegian background. The person-time for the Cox regression analyses was calculated from the start of follow-up, i.e., 2008 or the year the individual turned 16, and followed up until the year of diagnosis under study, death, age 30, or 2022, whichever came first. Those who died between 2008 and the start of follow-up were excluded (e.g. if a person was alive in 2008 but died before turning 16 and was included in follow-up). Individuals who died before the start of the follow-up period or before receiving a diagnosis were excluded. The analyses adjusted for sex, year of birth and parental education. We repeated the analyses, stratifying by parental region of origin for Fig. 3 and by sex in Supplementary File 1, Fig. 1. All analyses were conducted in Stata version 18.

The study was approved by the Regional Ethics Committee South-East (REK 2019/1286), with exemption from informed consent from participants as it is based on pseudo-anonymized register data and does not involve identifiable human or animal subjects.

## Results

Table 1 shows descriptive statistics of the sampled individuals by parental region of origin. Of the total sample (N = 1,529,755), 4.2% had two immigrant parents, 7.3% had one immigrant parent, and the remaining 88.5% had two Norwegian-born parents. Regarding parental region of origin, Asia was the parental region of origin for the majority (62%) of individuals with two immigrant parents, followed by Africa (16%) and non-EU European countries (10.5%). Conversely, over 65% of individuals with one immigrant parent predominantly had a background from the EU, USA, Canada, or Oceania, followed by Asia, with 21.5% having a mother from Asia and 15% having a father from Asia. Parental level of education was lower among individuals with two immigrant parents than the other groups, with the lowest attainment of education observed among parents from Africa and Asia. In the included samples of descendants, there were more males than females. Over 60% of individuals with one or two immigrant parents were born after the 1990s. Table 2 shows the numbers and percentages of individuals who received a mental disorder diagnosis across parental immigrant background.

**Table 1:** Sample characteristics of Norwegian-born children to one or two immigrant parents, aged 16–30 years, born between 1978 and 2006 and their parental region of origin. N (%)

	Two Norwegian-born parents	Two immigrant-born parents	EU, EEA, USA, Canada, Oceania	Europe (non-EU)	Asia	Africa	Latin America
	(N = 1,354,101)	(N = 63,849)	(N = 5,547)	(N = 6,707)	(N = 39,630)	(N = 10,169)	(N = 1,796)
Sex							
Male	695,877 (51.4)	32,957 (51.6)	2,882 (51.9)	3,403 (50.8)	20,472 (51.7)	5,226 (51.4	974 (54.2)
Female	658,224 (48.6)	30,892 (48.4)	2,665 (48.1)	3,304 (49.2)	19,158 (48.3)	4,943 (48.6)	822 (45.8)
Year of birth							
1978–1982	224,774 (16.6)	3,106 (4.9)	435 (7.8)	110 (1.6)	2,200 (5.5)	285 (2.8)	76 (4.2)
1983–1987	223,362 (16.5)	4,558 (7.1)	579 (10.4)	103 (1.5)	3,318 (8.4)	424 (4.2)	134 (7.5)
1988–1992	251,697 (18.6)	8,952 (14.0)	774 (13.9)	441 (6.6)	6,143 (15.5)	1,065 (10.5)	529 (29.5)
1993–1997	248,93 (18.4)	12,904 (20.2)	831 (15.0)	1,506 (22.5)	8,217 (20.7)	1,933 (19.0)	417 (23.2)
1998–2002	231,008 (17.1)	16,933 (26.5)	1,311 (23.7)	2,071 (30.9)	10,290 (26.0)	2,897 (28.5)	364 (20.3)
2003–2006	174,338 (12.9)	17,396 (27.3)	1,617 (29.2)	2,476 (36.9)	9,462 (23.9)	3,565 (35.1)	276 (15.4)
Primary school	90,991 (6.7)	19,126 (29.9)	354 (6.4)	1,227 (18.3)	13,859 (35.0)	3,438 (33.8)	248 (13.8)
High school	617,430 (45.6)	22,617 (35.4)	1,371 (24.7)	3,104 (46.3)	13,788 (34.8)	3,635 (35.8)	720 (40.1)
University (low)	466,759 (34.5)	15,441 (24.2)	1,855 (33.4)	1,728 (25.7)	9,024 (22.8)	2,234 (22.0)	600 (33.4)
University (High)	178,921 (13.2)	6,665 (10.4)	1,967 (35.5)	648 (9.7)	2,960 (7.5)	862 (8.5)	228 (12.7)

	Immigrant mother	EU, EEA, USA, Canada, Oceania	Europe (non-EU)	Asia	Africa	Latin America
	(N = 54,258)	(N = 35,461)	(N = 2,170)	(N = 11,682)	(N = 1,742)	(N = 3,203)
Sex						
Male	28,259 (52.1	18,546 (52.3)	1,116 (51.4)	6,014 (51.5)	908 (52.2)	1,675 (52.3)
Female	25,997 (47.9)	16,915 (47.7)	1,054 (48.6)	5,668 (48.5)	834 (47.8)	1,528 (47.7)
Year of birth						
1978–1982	5,397 (9.9)	4,650 (13.1)	30 (1.4)	428 (3.7)	104 (6.0)	185 (5.8)
1983–1987	6,003 (11.1)	4,701 (13.3)	36 (1.7)	885 (7.6)	140 (8.0)	241 (7.5)
1988–1992	8,282 (15.3)	6,018 (17.0)	69 (3.2)	1,547 (13.2)	221 (12.7)	427 (13.3)
1993–1997	9,699 (17.9)	6,705 (18.9)	253 (11.6)	1,929 (16.5)	303 (17.4)	509 (15.9)
1998–2002	12,059 (22.2)	7,094 (20.0)	704 (32.4)	3,001 (25.7)	460 (26.4)	800 (25.0)
2003–2006	12,818 (23.6)	6,293 (17.7)	1,078 (49.7)	3,892 (33.3)	514 (29.6)	1,041 (32.5)
Parental Education						
Primary school	3,375 (6.2)	1,435 (4.0)	133 (6.1)	1,435 (12.3)	130 (7.4)	242 (7.6)
High school	17,524 (32.3)	10,236 (28.9)	593 (27.3)	4,907 (42.0)	615 (35.4)	1,173 (36.6)
University (low)	19,957 (36.8)	13,720 (38.7)	762 (35.2)	3,740 (32.0)	660 (37.8)	1,075 (33.6)
University (High)	13,402 (24.7)	10,070 (28.4)	682 (31.4)	1,600 (13.7)	337 (19.4)	713 (22.3)

	Immigrant father	EU, EEA, USA, Canada, Oceania	Europe (non-EU)	Asia	Africa	Latin America
	(N = 57,547)	(N = 37,823)	(N = 1,623)	(N = 9,077)	(N = 5,733)	(N = 3,291)
Sex						
Male	29,794 (51.8	19,584 (51.8)	851 (52.4)	4,701 (51.8)	2,923 (51.0)	1,735 (52.7)
Female	27,753 (48.2)	18,239 (48.2)	772 (47.6)	4,376 (48.2)	2,810 (49.0)	1,556 (47.3)
Year of birth						
1978–1982	5,980 (10.4)	4,632 (12.2)	85 (5.2)	612 (6.7)	408 (7.1)	243 (7.4)
1983–1987	7,054 (12.3)	5,250 (13.9)	91 (5.6)	879 (9.7)	562 (9.8)	272 (8.3)
1988–1992	10,361 (18.0)	6,624 (17.5)	272 (16.7)	1,572 (17.3)	1,272 (22.2)	621 (18.9)
1993–1997	10,874 (18.9)	6,732 (17.8)	381 (23.5)	1,797 (19.8)	1,269 (22.1)	695 (21.1)
1998–2002	12,177 (21.2)	7,726 (20.4)	402 (24.7)	2,164 (23.8)	1,160 (20.2)	725 (22.0)
2003–2006	11,101 (19.3)	6,859 (18.1)	392 (24.3)	2,053 (22.6)	1,062 (18.5)	735 (22.3)
Parental Education						
Primary school	4,886 (8.5)	2,322 (6.1)	194 (11.9)	1,440 (15.9)	633 (11.0)	297 (9.0)
High school	18,829 (32.7)	11,929 (31.5)	748 (46.0)	3,344 (36.8)	1,673 (29.2)	1,135 (34.5)
University (low)	22,009 (38.3)	14,829 (39.2)	545 (33.7)	2,989 (32.9)	2,429 (42.4)	1,217 (37.0)
University (High)	11,823 (20.5)	8,743 (23.1)	136 (8.4)	1,304 (14.4)	998 (17.4)	642 (19.5)

**Table 2:** Mental disorder diagnoses given in specialist healthcare between the years 2008 and 2022 among those aged 16–30 years, born in Norway with two Norwegian-born parents, two immigrant parents, and one Norwegian and immigrant parent (mother/father). N (%)

	Norwegian-born parents (N = 1,352,101)	Immigrant-born parents (N = 63,849)	EU, EEA, USA, Canada, Oceania (N = 5,547)	Europe (non-EU) (N = 6,707)	Asia (N = 39,630)	Africa (N = 10,169)	Lati America (N = 1,796)
Diagnoses							
Any mental disorder	236,906 (17.4)	8, 578 (13.42) ***	929 (16.8) ***	779 (11.6) ***	5,057 (12.8) ***	1,373 (13.5)	440 (24.5) ***
Depression	98,602 (7.3)	3,730 (5.84) ***	399 (7.2) ***	301 (4.5) ***	2,317 (5.9)	503 (4.9) ***	210 (11.7) ***
Anxiety	142,083 (10.5)	5,157 (8.07) ***	523 (9.4) ***	501 (7.5) *	3,044 (7.7) ***	800 (7.9)	289 (16.1) ***
Bipolar	11,496 (0.9)	309 (0.48) ***	47 (0.9) ***	24 (0.4)	162 (0.4) ***	60 (0.6)	16 (0.9) **
Eating disorder	17,775 (1.31)	505 (0.79) ***	86 (1.6) ***	56 (0.8)	279 (0.7) **	60 (0.6) **	24(1.3) **
Schizophrenia	9,582 (0.71)	655 (1.03) ***	49 (0.9)	34 (0.5) ***	382 (1.0) *	174 (1.7) ***	16 (0.9)

	Immigrant mother (N = 54,258)	EU, EEA, USA, Canada, Oceania (N = 35,461)	Europe (non-EU) (N = 2,170)	Asia (N = 11,682)	Africa (N = 1,742)	Latin America (N = 3,203)
Diagnoses						
Any mental disorder	10,085 (18.6) ***	6,478 (18.3) ***	433 (20.0)	2,135 (18.3) ***	340 (19.5)	699 (21.8)
Depression	4,193 (7.7) ***	2,716 (7.7) ****	166 (7.7)	902 (7.7) ***	143 (8.2)	266 (8.3)
Anxiety	5,888 (10.9) **	3,780 (10.7) ***	243 (11.2)	1,285 (11.0) ***	193 (11.1)	386 (12.1)
Bipolar	471 (0.9)	334 (0.9)	13 (0.6)	86 (0.7) **	15 (0.9)	23 (0.7)
Eating disorder	730 (1.4)	521 (1.5) *	32 (1.5)	122 (1.0) ***	29 (1.7)	26 (0.8) **
Schizophrenia	503 (0.9) ***	295 (0.8) ***	16 (0.7)	118 (1.0)	25 (1.4)	49 (1.5) **

	Immigrant father (N = 57,547)	EU, EEA, USA, Canada, Oceania (N = 37,823)	Europe (non-EU) (N = 1,623)	Asia (N = 9,077)	Africa (N = 5,735)	Latin America (N = 3,291)
Diagnoses						
Any mental disorder	12,837 (22.3) ***	7,812 (20.7)	422 (26.0) ***	2,215 (24.4) ***	1,517 (26.5) ***	871 (26.5) ***
Depression	5,439 (9.5) ***	3,274 (8.7)	173 (10.6) **	964 (10.6) ***	667 (11.6) ***	361 (11.0) ***
Anxiety	7,724 (13.4) ***	4,670 (12.4)	285 (17.6) ***	1,330 (14.7) ***	909 (15.9) ***	530 (16.1) ***
Bipolar	645 (1.1) ***	411 (1.1) *	14 (0.9)	106 (1.2)	80 (1.4) **	34 (1.0)
Eating disorder	944 (1.6) ***	575 (1.5)	29 (1.8)	165 (1.8) **	111 (1.9) **	64 (1.9) *
Schizophrenia	633 (1.1) ***	351 (0.9) *	18 (1.1)	126 (1.4) ***	99 (1.7) ***	39 (1.9) **

**Fig 2:**
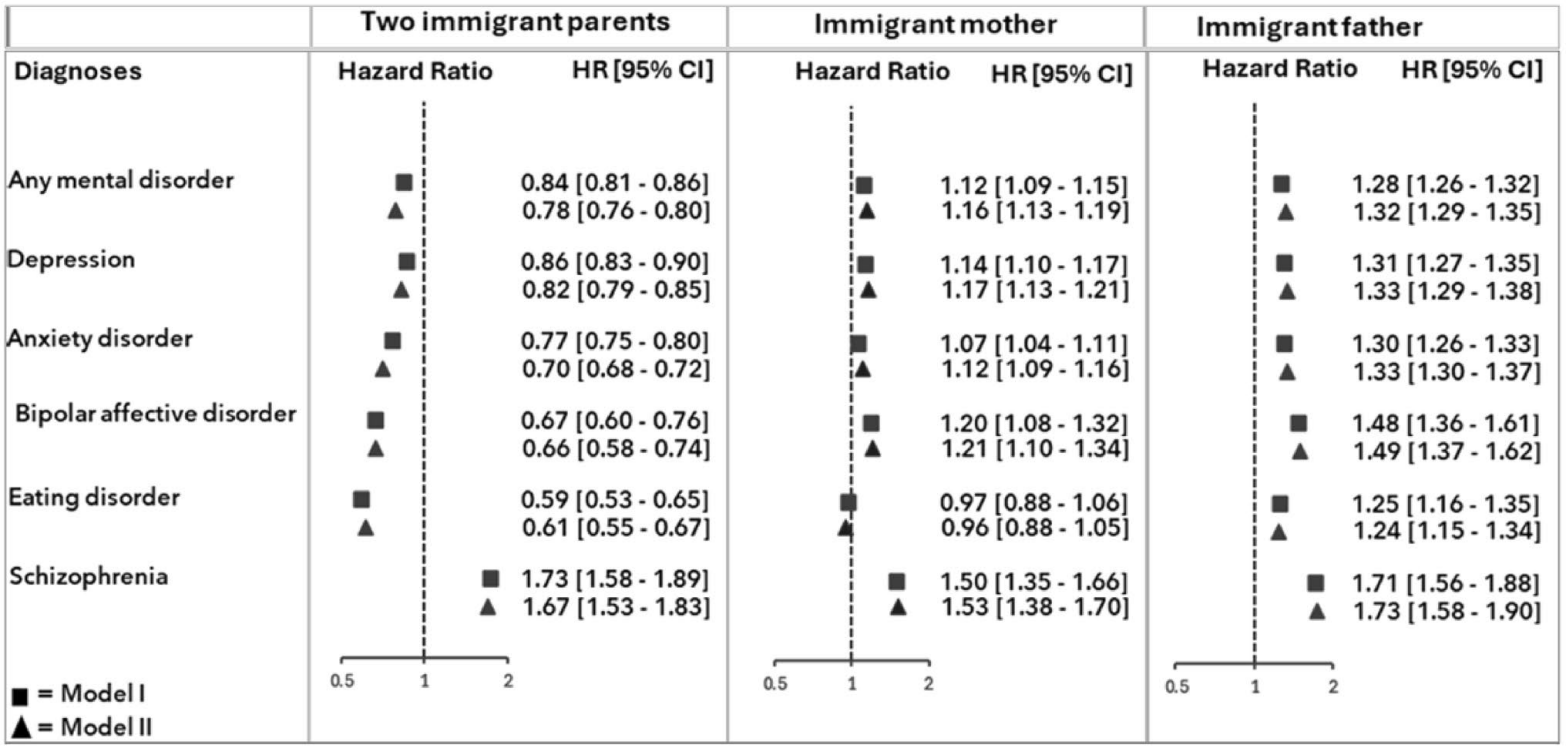
Hazard ratios (95% confidence intervals) receiving diagnoses of mental disorders given in specialist healthcare between 2008 and 2022 among children aged 16–30 years of Norwegian-born to one or two immigrant parents (reference group: Norwegian-born parents). Model I: Adjusted for sex and year odf birth. Model II: Adjusted foer sex, birth year and parental education

As demonstrated in the Cox regression analysis results presented in Fig. 2, the hazard of receiving a diagnosis of any mental health condition [0.84 (95% CI: 0.81– 0.86)], as well as of depression, anxiety, bipolar affective disorder, and eating disorder, was statistically significantly lower among youth and young adults with two immigrant parents compared to those with Norwegian background. Only the hazard of a schizophrenia diagnosis was statistically significantly higher [1.73 (95% CI: 1.58–1.89)]. Among those with one immigrant parent, the hazard of any mental disorder diagnosis was statistically significant higher for both those with an immigrant mother [1.12 (95% CI: 1.09–1.15)] and those with an immigrant father [1.28 (95% CI: 1.26–1.32)] compared to those with a Norwegian background, with the exception of eating disorders among those with an immigrant mother (Fig. 2). The estimates did not change notably with the adjustment for parental education (model II in Fig. 2).

**Fig 3:**
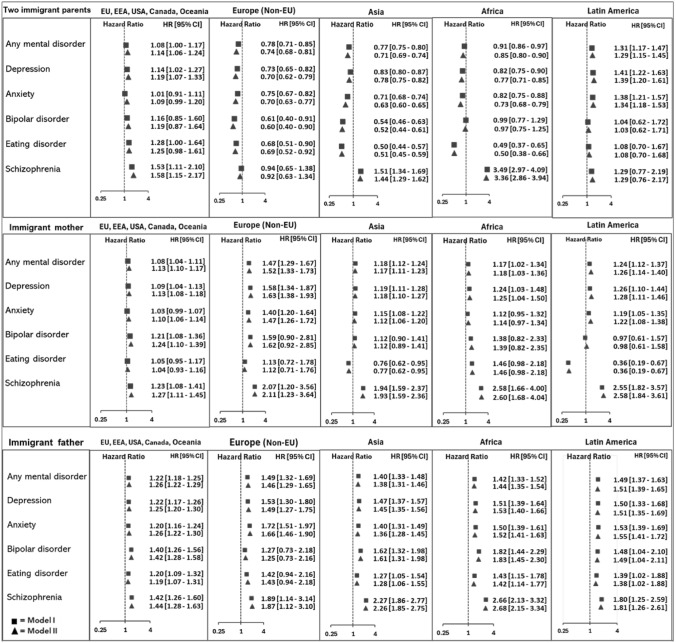
Hazard ratios (95% confidence intervals receiving diagnoses of mental disorders given in specialist healthcare between 2008 and 2022 among children aged 16–30 years of Norwegian-born to one or two immigrant parents (reference group: Norwegian-born parents) by parental region of origin. Model I: Adjusted for sex and year of birth. Model II: Adjusted for sex, birth year, and parental education

We also found statistically significant variations in mental health risks based on the parental region of origin (Fig. 3). Compared to those with a Norwegian background, individuals with two parents from Latin America had the highest risk of being diagnosed with most mental disorders, followed by those with parents from the EU, EEA, USA, Canada, Oceania, Asia, and Africa. However, those with one Norwegian parent and one immigrant parent, particularly from Latin America, Asia, or Africa, also showed elevated risks. Estimates were generally the same stratified by sex, however, the increased risk for schizophrenia among males with two immigrant parents is larger than the increased risk for females with two immigrant parents (supplementary file, Fig. 1).

## Discussion

This study found lower hazards of receiving mental disorder diagnoses in specialist health care among youth and young adults with two immigrant parents compared to those with two Norwegian-born parents, but higher hazards among those with one immigrant parent. Regarding the parental region of origin, individuals with two immigrant parents from Asia, Africa, or Latin America consistently had lower hazards across most diagnoses, whereas hazards were higher when both parents were from the EU, EEA, USA, Canada, or Oceania. Among those with one immigrant parent from most regions, there were higher hazards of receiving mental health disorder diagnoses, particularly when the immigrant parent was the father.

That youth and young adults with two immigrant parents have a lower risk of receiving a mental disorder diagnosis in specialist health care than their peers with a non-immigrant background is in line with existing research from Norway [13], Finland [10], and Denmark [11, 12]. It contrasts, however, with findings on the risk of hospitalisation for non-affective disorders in Denmark [23]. As data from surveys indicate that descendants report higher levels of psychological distress, anxiety, and depression [8, 9, 14, 24] than native-background peers, it is probable that challenges related to underutilisation of services are at least partly the cause of the lower hazards found in this study. For immigrants, there may be several barriers to the use of mental health care, such as poor language proficiency, lack of system knowledge, and cultural perceptions of mental disorders and related stigma. Although such structural barriers to health care can be lower among descendants in early adulthood, parental influence, reluctance to discuss mental health issues, and cultural stigma may represent barriers to seeking and adhering to mental health care [25, 26]. Barriers to mental health care could be anticipated to be lower among descendants from EU/EEA/USA/Canada/Oceania, which is reflected in slightly higher hazards of mental disorder diagnoses among descendants from this region. Additionally, cultural identity, lack of trust, and past negative encounters with mental health practitioners can also inhibit help-seeking [25, 26], leading to underdiagnosis. It is also possible that our findings reflect a lower prevalence of mental health disorders among descendants than native youth. Possible explanations could include close family ties and a strong sense of belonging to a cultural group. A study reported better psychological health via greater closeness to family members and perceived social support [27], although previous studies report higher rates of self-reported mental health complaints [8, 9, 14, 24]. A Final explanation is that general practitioners, who often serve as gatekeepers to specialist health care, may be less likely to recognise mental disorders in young people with immigrant backgrounds or refer them less frequently to specialist services compared to their peers with native-born parents [28].

Noteworthy is the higher risk of a diagnosis of schizophrenia among those with both one and two immigrant parents, compared to those with a Norwegian background, with more pronounced differences for males than females. In line with this, several studies have reported a higher hazard of schizophrenia among descendants [16, 29–31], and particularly those of non-Western background [32]. Moreover, inpatient treatment and emergency room contacts related to a schizophrenia diagnosis are higher among male, but not female, descendants compared to native peers in Denmark [12]. As schizophrenia has a severe nature and more overt symptoms than other mental health disorders, it is more likely to be recognised and, eventually, addressed by healthcare services, irrespective of barriers to accessing mental healthcare. A similar situation is found regarding autism spectrum disorders (ASD), where children of immigrants have a higher risk of an early diagnosis, indicating a severe form of ASD [33]. Besides, studies show that males are more prone than females to externalising disorders and higher suicide rates [18, 31, 34–36], and our results suggest that the risk of schizophrenia is more elevated among descendant males than females, compared to those with no immigrant background.

Youth and young adults with one immigrant parent had a higher risk of mental disorder diagnoses than Norwegian background peers, in line with previous findings [10, 11, 13, 14, 32]. Being raised in a family of two cultures can be linked to skills in adapting and resilience [10], as well as better socioeconomic conditions than families with two immigrant parents. However, it may also lead to struggles with identity and double social pressures, causing poorer mental health [10]. Moreover, intercultural marriages are often linked to higher levels of conflict and divorce [37, 38], which can also negatively impact the mental health of the children. The differences in risk of diagnoses for mental disorders compared to Norwegian background youth and young adults were larger for those with an immigrant father (a Norwegian mother) than an immigrant mother (a Norwegian father). A study from Finland reported similar results [10]. This may suggest that the father’s migration experiences, employment difficulties, lower SEP, experiences of discrimination, and stress could more negatively impact family dynamics and the youth’s mental health than the mother’s. Moreover, as mothers often take more responsibility for children`s health and well-being than fathers, those with a non-immigrant mother may experience fewer structural and cultural barriers to seeking mental health care, resulting in better access to care when required.

## Strengths and Limitations

The study’s strengths included the use of a large, nationally representative sample, with diagnoses from specialist healthcare from 2008 to 2022. We did not have access to data from primary care services, which could have nuanced the picture regarding early diagnoses, follow-up and treatment. In Norway, the general practitioner is the gatekeeper to specialist healthcare, and our findings may thus, to some extent, reflect the use of primary care. We cannot conclude to what extent our results reflect differences in the prevalence of mental health disorders or in the use of specialist healthcare services. Neurodevelopmental disorders were excluded, as these conditions typically occur during early childhood, and we did not have data to assess diagnoses given before 2008, i.e. earlier in life for most of the population. We neither had access to data on substance use disorders, although including these diagnoses could have given a broader picture of mental health issues in this population. Further studies are warranted to investigate potential barriers to mental health services among young descendants.

## Conclusion

Youth and young adults with two immigrant parents had a lower, and those with one immigrant parent had a higher, risk of receiving a mental disorder diagnosis in specialist health care. The results indicate that descendants of two immigrant parents may face barriers to mental health care. Future research should investigate such barriers and ways to overcome them. Our study also suggests that youth and young adults with one immigrant parent, especially with an immigrant father, are at higher risk of mental disorders. This highlights the need for targeted interventions to prevent mental disorders in these groups and to address unmet mental health needs.

## Supplementary Information

Below is the link to the electronic supplementary material.

Supplementary file1 (JPG 239 KB)

## Data Availability

Due to data regulations, the datasets generated and analysed during this study are not publicly available but can be obtained from the registry owners.
