# Supplementary figures and images for: Diagnoses of Mental Health Disorders Among Norwegian-Born Youth and Young Adults with Immigrant Parents—A Register-Based Study
Source: J Immigr Minor Health. 2025 Jul 9;27(5):667–76. doi: 10.1007/s10903-025-01726-6 (PMC12420709; doi:10.1007/s10903-025-01726-6)

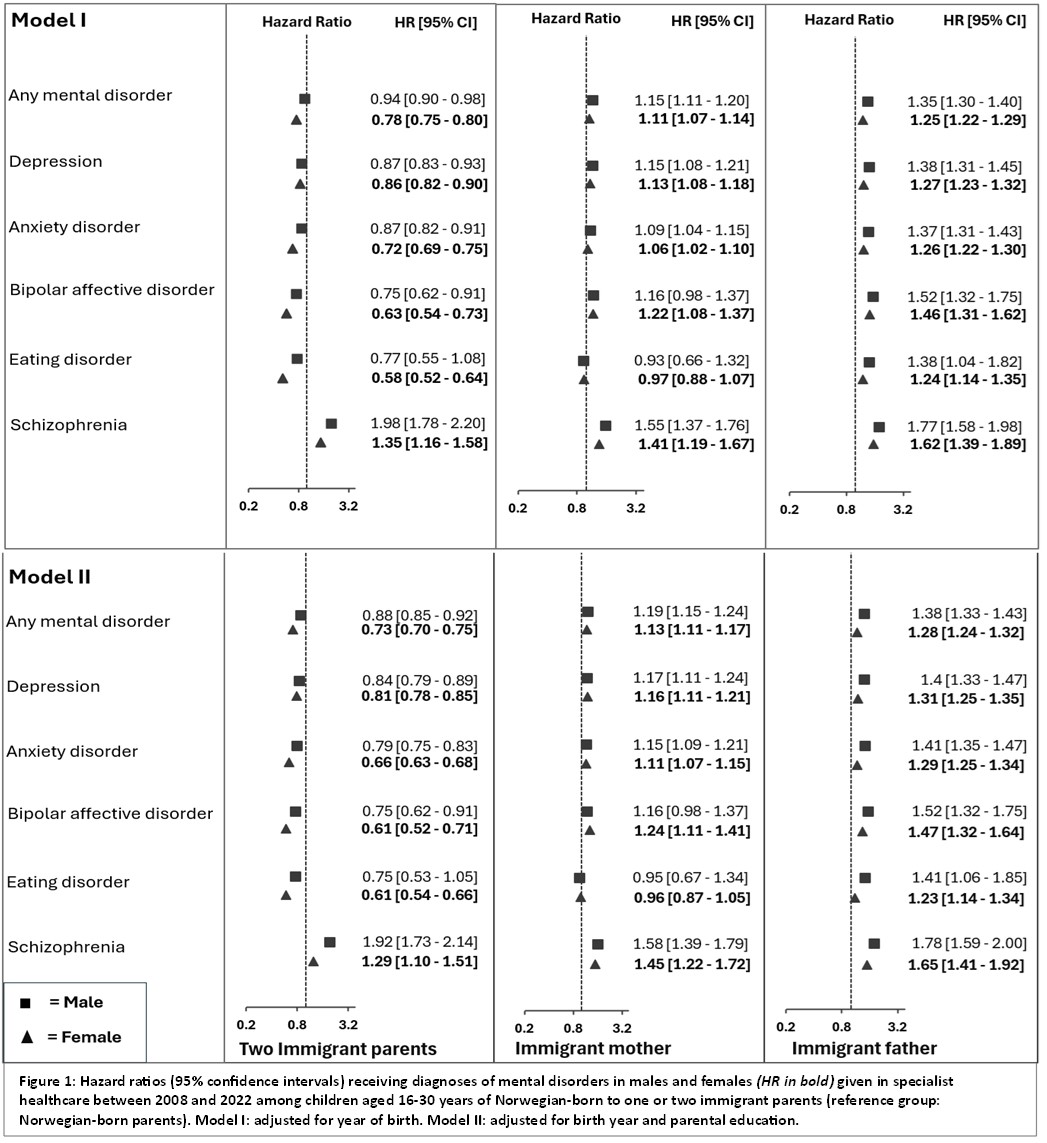

Supplement: Supplementary file 1 — Supplementary file1 (JPG 239 KB) [file 10903_2025_1726_MOESM1_ESM.jpg]
